# Quercetin attenuates cisplatin-induced fatigue through mechanisms associated with the regulation of the HPA axis and MCP-1 signaling

**DOI:** 10.3389/fnut.2025.1530132

**Published:** 2025-01-30

**Authors:** Cheng-Hung Chuang, Yu-An Tai, Ting-Jing Wu, Ying-Jui Ho, Shu-Lan Yeh

**Affiliations:** ^1^Department of Nutrition, Hungkuang University, Taichung, Taiwan; ^2^Department of Nutritional Science, Chung Shan Medical University, Taichung, Taiwan; ^3^Institute of Medicine, Chung Shan Medical University, Taichung, Taiwan; ^4^Department of Psychology, Chung Shan Medical University, Taichung, Taiwan; ^5^Department of Nutrition, Chung Shan Medical University Hospital, Taichung, Taiwan

**Keywords:** quercetin, cisplatin, cancer-related fatigue, HPA axis, MCP-1

## Abstract

**Introduction:**

Cancer-related fatigue (CRF) is a common symptom induced by chemotherapy. The main objective of the present study was to investigate whether quercetin regulates the hypothalamic-pituitary-adrenal (HPA) axis and chemoattractant protein-1 (MCP-1) signaling, two factors contributing to CRF in mice exposed to cisplatin.

**Methods:**

Male BALB/c mice were randomly assigned to the following five groups for 15 weeks: Control, CDDP, CDDP+TAK779 (an antagonist of MCP-1 receptor, human CC chemokine receptor R2 (CCR2)), CDDP+OQ (a diet containing 1% quercetin) and CDDP+IQ (quercetin given by ip, 10 mg/kg, 3 times/week).

**Results:**

The results first showed that OQ and IQ significantly increased grip strength and locomotor activity, decreased plasma cortisol/corticosterone levels, and decreased the corticotropin releasing hormone (CRH) mRNA level in the brain tissues in mice exposed to CDDP. OQ and IQ also decreased CDDP-induced plasma levels of MCP-1 as well as the mRNA expression of MCP-1 and CCR2 in the brain stem. TAK779 significantly increased grip strength and tended to decrease the cortisol/corticosterone levels in CDDP-exposed mice, indicating the association between the HPA axis and MCP-1 signaling.

**Conclusion:**

Taken together, the study suggests that quercetin could attenuate CDDP-induced CRF through the mechanisms associated with downregulation of the HPA axis and MCP-1 signaling in mice.

## Introduction

Cancer-related fatigue (CRF) is a common and weakening symptom in cancer patients induced by the tumor itself or its treatment, including chemotherapy (1). Because the sense of tiredness persists even after rest, CRF diminishes both physical and psychological functioning, decreases the quality of life, and potentially increases morbidity and mortality in cancer patients (2). The possible factors contributing to CRF is complex, including mood disturbances, anemia, malnutrition, muscle atrophy (3) and central nervous system dysfunctions such as disruptions in the hypothalamic–pituitary–adrenal (HPA) axis and increase of pro-inflammatory cytokines (4). Many strategies, including exercise, psychological education, and pharmacological and nutritional interventions have been suggested to attenuate CRF (4). However, effective treatments for CRF remain limited and further studies are needed to explore novel therapeutic strategies.

Glucocorticoids, also called stress hormones, released from the activated HPA axis, play a vital role in the neuroendocrine system and are required for stress adaptation (5, 6). The major structures within the HPA axis involve the hypothalamus, pituitary, and adrenal glands. First, the hypothalamus releases corticotropin releasing hormone (CRH) that in turn stimulates the pituitary to produce proopiomelanocortin (POMC). POMC, then, converts into adrenocorticotropic hormone (ACTH) that is secreted into the systemic circulation and acts at the adrenal gland to stimulate the synthesis and secretion of glucocorticoids, including cortisol and cortisone (6). A proper increase of the levels of glucocorticoids is important for stress response, however, dysregulation or prolonged HPA axis activation is energetically costly and is associated with numerous physiological and psychological disease states such as chronic fatigue syndrome including CRF (7, 8).

In addition, the increase of monocyte chemoattractant protein-1 (MCP-1), also called chemokine ligand 2 (CCL2), level may be another risk factor for CRF (9). MCP-1 is a chemokine that regulates the movement and infiltration of monocytes/macrophages and plays an important role in inflammation in both the peripheral and central nervous systems. Studies have confirmed that the induction of MCP-1 and its receptor, human CC chemokine receptor R2 (CCR2), is associated with the development of many diseases (10). Recent clinical and animal studies have shown that elevated serum MCP-1 is associated with CRF induced by cancer itself or chemotherapy (9, 11).

Our previous studies have shown that quercetin, a flavonoid that is commonly present in plant foods and possesses many bioactivities including antioxidant activity (12, 13) improves grip strength and locomotor activity, indicators of fatigue, partly through attenuating the muscle loss in mice exposed to CDDP (14). CDDP treatment may also induce CRF by disruption of the central nervous system, that is, increasing the plasma level of cortisol (15) and MCP-1 (14), while quercetin and its glycoside derivative have been shown to exert antidepressant and antianxiety effects by improving hypothalamic–pituitary–adrenal (HPA) axis dysfunction, and reducing inflammatory states (16, 17). We, therefore, conducted an animal study to investigate whether quercetin regulates the HPA axis as well as the expression of MCP-1 and its receptor C-C chemokine receptor type 2 (CCR2) in the brain in mice exposed to CDDP. We also used TAK-779 (*N*,*N*-dimethyl-*N*-[4-[[[2-(4-methylphenyl)-6,7-dihydro-5*H*-benzocyclohepten-8-yl]carbonyl]amino]benzyl]-tetrahydro-2*H*-pyran-4-aminium chloride), an antagonist of CCR2 (18, 19), as a positive control for MCP-1/CCR2 signaling.

## Materials and methods

### Reagents

All chemicals used were of reagent grade or higher. Quercetin was obtained from Alfa Aesar (Tewksbury, MA, USA). CDDP was obtained from Acros Organics (Geel, Belgium). TAK779 was from AdooQ Bioscience (Irvine, CA, USA).

### Animal study

Male BALB/c mice aged 4 weeks were acquired from the National Laboratory Animal Center (Taipei, Taiwan) and were housed in an animal room with an alternating 12-h light/dark cycle, controlled temperature (25°C) and humidity (50–60%). After being acclimated for 2 weeks, the animals were randomly assigned to the following five groups (*n* = 6–7/group) for 15 weeks: Control, CDDP alone, CDDP+TAK779 (an antagonist of MCP-1 receptor, CCR2), CDDP+OQ (quercetin given by a diet containing 1% quercetin) and CDDP+IQ (quercetin given by intraperitoneal injection (ip), 10 mg/kg, 3 times per week). The doses of quercetin and CDDP were used according to the previous study (20). TAK779 was given by ip at 6 mg/kg, 3 times/week (21). All animals were allowed free access to a standard rodent diet (Lab 5,001, Purina Mills, St. Louis, MO), or 1% quercetin supplemented diet and water during the study. Body weight and food intake of the animals were recorded weekly during the experiment. The animals were cared for and sacrificed according to the method described previously (14). After being sacrificed, blood samples, muscle tissues and brain tissues were collected and stored at −80°C until analysis. All study protocols were approved by the Institutional Animal Care and Use Committee of Chung Shan Medical University (IACUC approval no. 2189).

### Locomotor activity and maximum grip strength (MGS)

At week 5, four mice in each group were individually housed in transparent cages (17 × 28 × 12.5 cm) after CDDP injection for 12 h, and locomotor activities were recorded (from 11:00 pm to 01:00 am). Movement and rest time spends were analyzed using the Video Trace Mouse II software (SINGA, Taiwan). Furthermore, the MGS of the forelimb of BALB/c mice was measured using a grip strength meter (Ugo Basile, Italy) after CDDP injection for 24 h during week 6 and 12. The details of the protocols were described previously (14).

### ELISA assay for cortisol, corticosterone, MCP-1 and proinflammatory cytokines levels

According to the instructions of enzyme-linked immunosorbent assay (ELISA) kits, the plasma levels of cortisol (KEG 008; R&D Systems, Minneapolis, MN, USA) and corticosterone (KEG 008; R&D Systems, Minneapolis, MN, USA), MCP-1 (DY479-05; R&D Systems, Minneapolis, MN, USA) were determined. In addition, the cerebrum tissues (0.03 g) were homogenized in 300 μL PBS containing protease inhibitor cocktail (cOmplete™, Roche, Mannhim, Germany) and phosphatase inhibitor cocktail (100 × phosphatase inhibitor, Goalbio, Taipei, Taiwan) by Tissuelyzer (Qiagen, Hilden, Germany) and then were subjected to ELISA kit (Invitrogen, Carlsbad, MA, USA) to determine proinflammatory cytokines, TNF-*α*, IL-6, and IL-1β, levels. Protein concentration of samples were determined by Protein Assay Dye Reagent Concentrate (Bio-Rad Laboratories, Hercules, CA, USA).

### Quantitative real-time PCR (qPT-PCR) for the mRNA levels of CRH, POMC, MCP-1 and CCR2

The mRNA levels of CRH in the hypothalamus, POMC in the pituitary as well as MCP-1 and CCR2 in the brainstem were determined by qPT-PCR. The RNA samples were isolated and quantitated according to the method described previously (21). In brief, brain tissue was lysed by using the mixture of 200 μL of TRIzol reagent (Invitrogen, Carlsbad, CA, USA), 800 μL of TRIzol, and 200 μL of chloroform. Isopropanol and 75% ethanol were used to precipitate and wash the mRNA pellet. The RNA concentration was then adjusted to approximately 2 or 1 μg/μL with diethyl pyrocarbonate solution. Furthermore, the High-Capacity cDNA Reverse Transcription Kit (Thermo Fisher Scientific, Richmond, VA, USA) was used for reverse transcription. Then, for qPT-PCR, Fast SYBR Green Master Mix (Thermo Fisher Scientific, Richmond, VA, USA) was prepared according to the manufacturer’s instructions. The primer sequences for determined genes are shown in Table 1. The mixture was incubated at 95°C for 20 s, followed by 40 cycles at 95°C for 1 s and 60°C for 20 s.

**Table 1:** The primer sequences of determined genes.

Gene name	Sequence	
CRH	Forward	5’-CCTGGGGAATCTCAACAGAA-3′
	Reverse	5’-AACACGCGGAAAAAGTTAGC-3′
POMC	Forward	5’-CATTAGGCTTGGAGCAGGTC-3’
	Reverse	5’-TCTTGATGATGGCGTTCTTG-3’
MCP-1	Forward	5’-TGATCCCAATGAGTAGGCTGGAG-3’
	Reverse	5’-ATGTCTGGACCCATTCCTTCTTG-3’
CCR2	Forward	5’-GAAGAGGGCATTGGATTCACC-3’
	Reverse	5’-TATGCCGTGGATGAACTGAGG-3’
GAPDH	Forward	5’-GTCGGTGTGAACGGATTTG-3’
	Reverse	5’-AAGATGGTGATGGGCTTCC-3’

### HPLC assay for the total quercetin level

The levels of total quercetin (including quercetin and its glucuronide/sulfate conjugates) in the brain tissues were measured using the HPLC method described previously (22). Briefly, homogenized tissue samples (0.03 g tissue in 300 μL PBS) were incubated with 5 μL of Helix pomatia enzyme mixture at 37°C for 2 h first. Then, the samples were deproteinized and centrifuged. The supernatant was filtered and analyzed by HPLC with spectrophotometric detection at 370 nm.

### Statistical analysis

All data are expressed as mean ± standard deviation (SD). Statistical analysis was performed using SPSS software, Vers. 18 (IBM, USA). The differences between the control group vs. the CDDP group and the CDDP group vs. each supplemented group were assessed by Student’s *t*-test. *p-*values <0.05 were considered statistically significant.

## Results

### Locomotor activity and MGS

**Figure 1:**
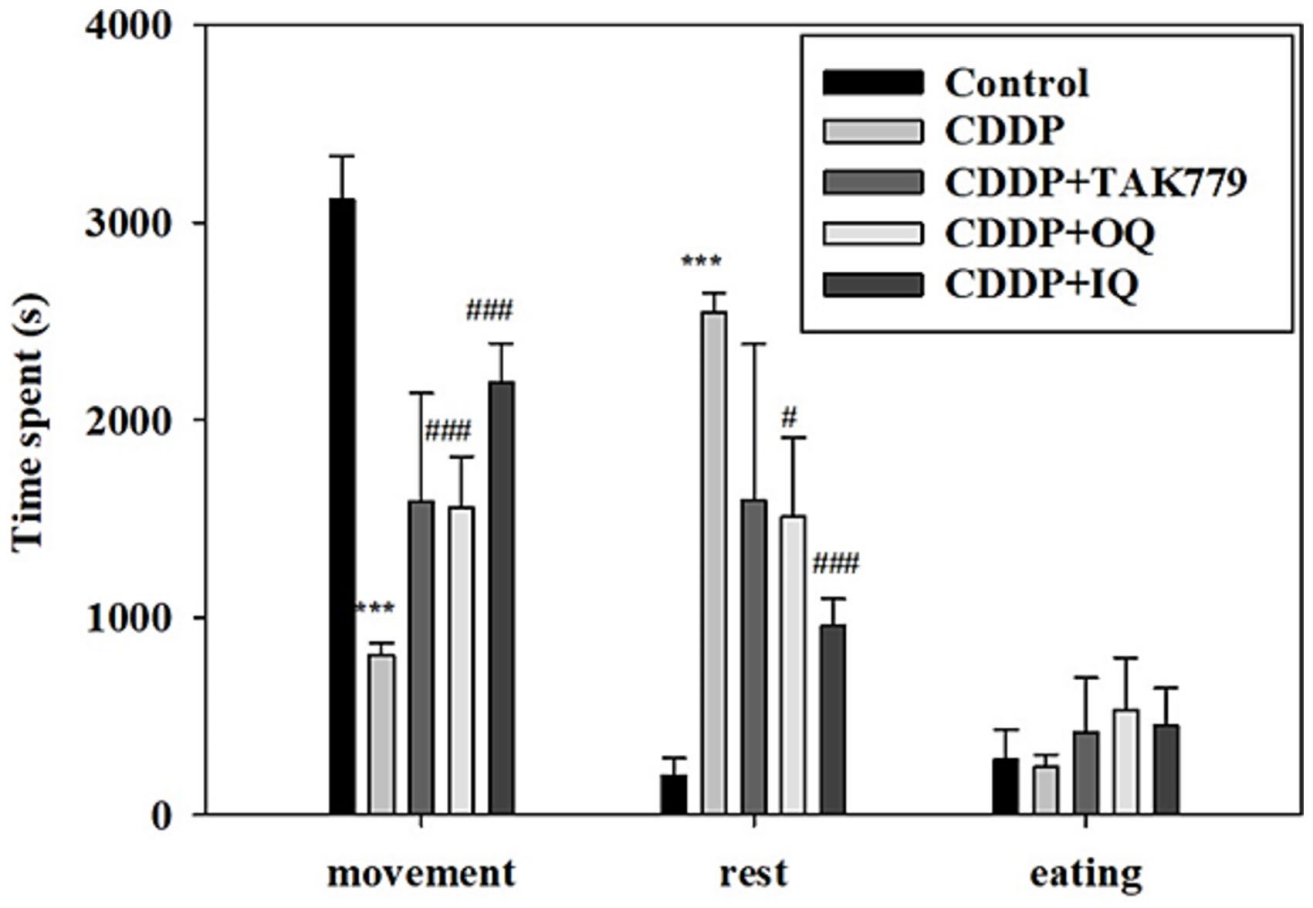
The effect of cisplatin (CDDP) alone or in combination with quercetin or TAK779 on the locomotor activities in BALB/c mice. Quercetin was administered by a diet containing 1% quercetin (OQ) or intraperitoneal injection (IQ). The control group received the control diet and vehicle only. Values are expressed as mean ± SD. *p < 0.05, **p < 0.01, ***p < 0.001 denote a significant difference from the control group; #*p* < 0.05, ##*p* < 0.01, ###*p* < 0.001 denote a significant difference from the CDDP group.

**Figure 2:**
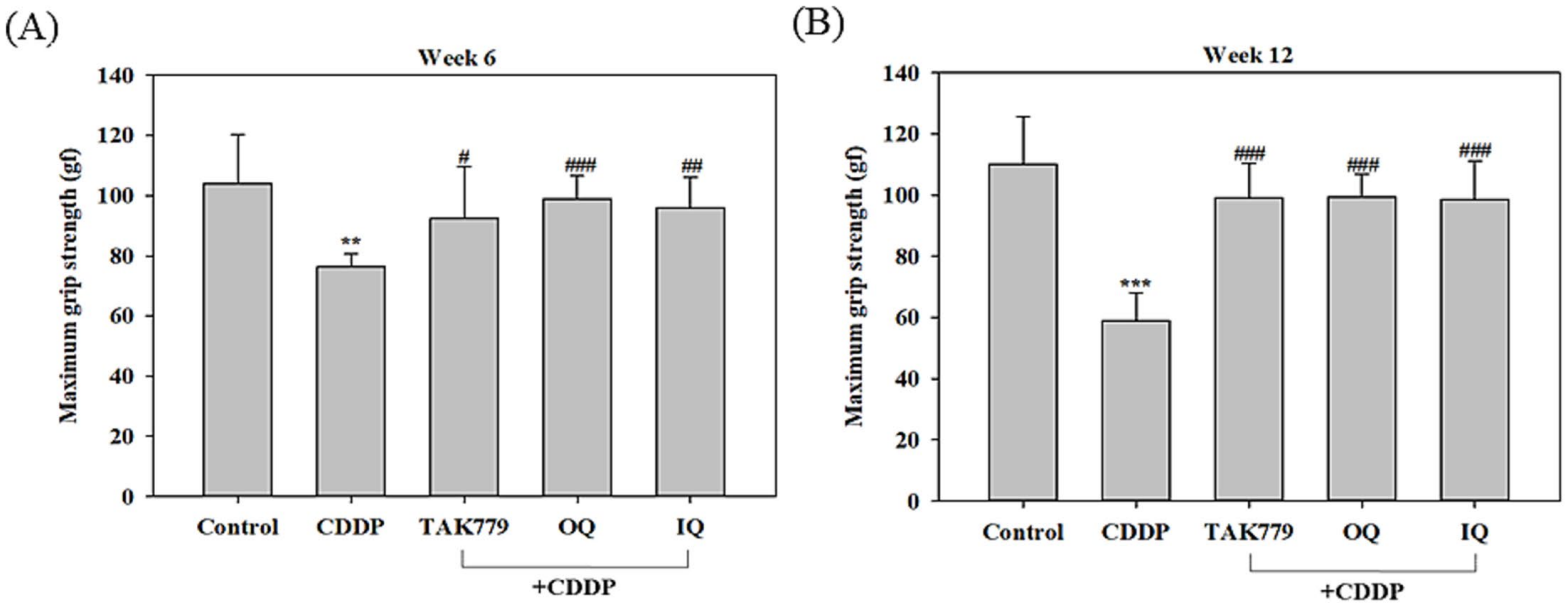
The effect of cisplatin (CDDP) alone or in combination with quercetin or TAK779 on the maximum grip strength at week 6 **(A)** and week 12 **(B)** in BALB/c mice. Quercetin was administered by a diet containing 1% quercetin (OQ) or intraperitoneal injection (IQ). The control group received the control diet and vehicle only. Values are expressed as mean ± SD. *p < 0.05, **p < 0.01, ***p < 0.001 denote significant differences from the control group; #*p* < 0.05, ##*p* < 0.01, ###*p* < 0.001 denote significant differences from the CDDP group.

We determined locomotor activity and MGS as markers of fatigue. As expected, the locomotor activity assay showed that CDDP significantly decreased the movement time and increased the rest time (Figure 1). Both OQ and IQ significantly attenuated the effects of CDDP. TAK779 tended to increase the time of movement but the effect was not significant. Consistently, CDDP also significantly decreased MGS at week 6 and week 12 by about 27 and 46%, respectively (Figure 2). OQ, IQ, and TAK779 all significantly restored MGS to levels similar to the control group.

### Body weight, food intake, and muscle weight

As shown in Table 2, at the end of the study the average body weight and food intake of mice in the CDDP group was significantly lower than that of the control group. OQ, IQ, and TAK779 did not significantly improve the body weight, or the food intake compared to the CDDP alone group. In addition, CDDP also significantly decreased the total weight of muscles (the sum of the triceps brachii, quadriceps femoris, and gastrocnemius muscles), while TAK779 and IQ significantly restored the effect of CDDP (*p* < 0.01).

**Table 2:** The effect of cisplatin (CDDP) alone or in combination with quercetin or TAK779 on body weight, food intake and total muscle weight in BALB/c mice.

Treatment	Control	CDDP	CDDP+TAK779	CDDP+OQ	CDDP+IQ
Body weight before CDDP injection (g)	23.1 ± 1.1	22.3 ± 1.1	22.2 ± 0.9	22.0 ± 1.4	22.5 ± 1.0
Body weight at the end of the experiment (g)	30.3 ± 1.6	17.6 ± 1.2^***^	18.5 ± 1.9	17.9 ± 1.2	18.0 ± 1.8
Food intake (g/day)	7.4 ± 1.0	5.8 ± 0.7^***^	6.6 ± 1.8	5.9 ± 0.8	6.2 ± 0.8
Total muscle weight (g)	1,077 ± 66	527 ± 61^***^	638 ± 67^##^	590 ± 82	633 ± 68^##^

Quercetin was administered by a diet containing 1% quercetin (OQ) or intraperitoneal injection (IQ). The control group received the control diet and vehicle only. Values are expressed as mean ± SD. *p < 0.05, **p < 0.01, ***p < 0.001 denote a significant difference from the control group; #*p* < 0.05, ##*p* < 0.01, ###*p* < 0.001 denote a significant difference from the CDDP group. Total muscle weight: the sum of tricepsquadriceps, quadriceps, and gastrocnemius muscle.

### Expression of key hormones in the HPA axis

**Figure 3:**
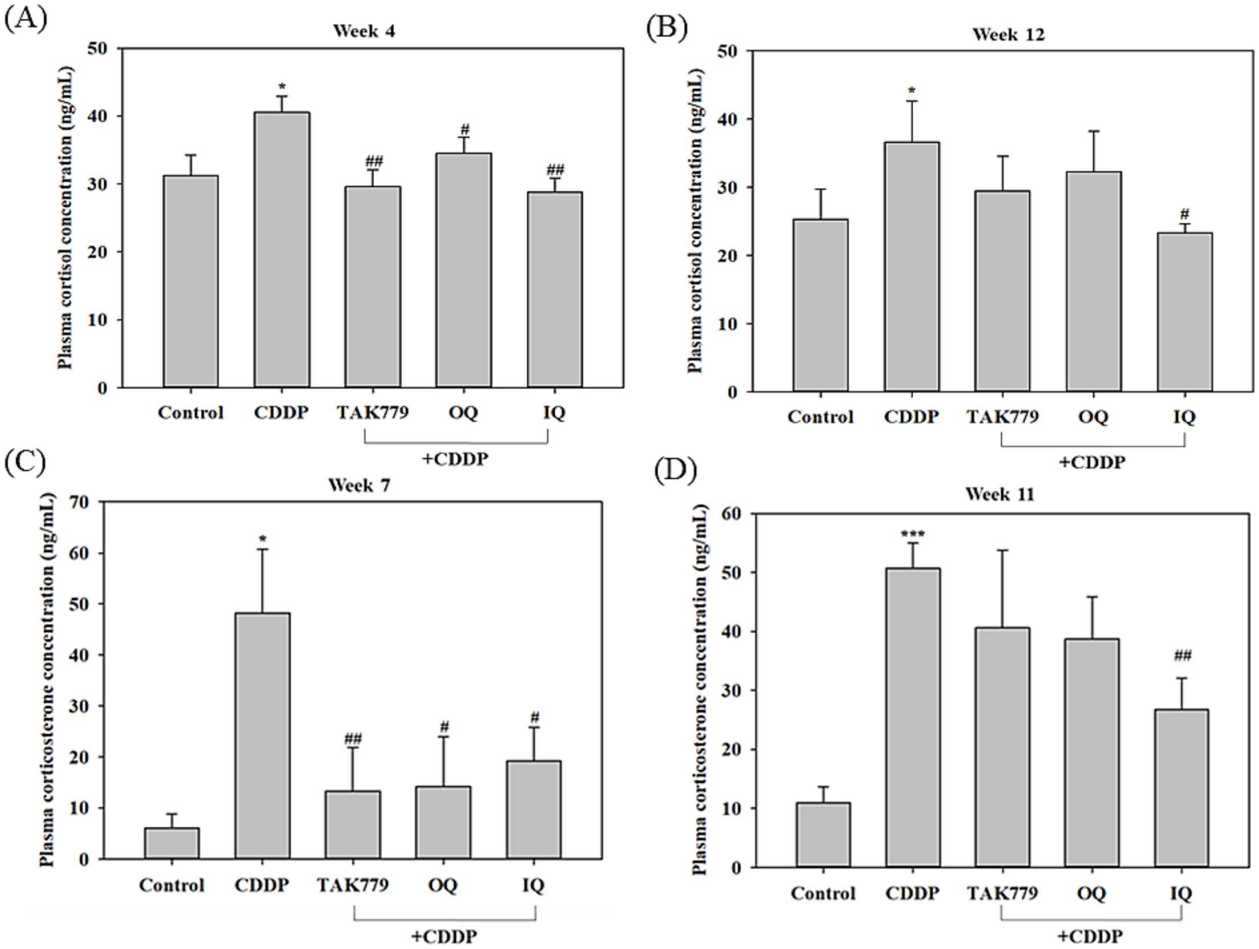
The effect of cisplatin (CDDP) alone or in combination with quercetin or TAK779 on the plasma levels of cortisol at week 4 and week 12 (**A,B**, respectively) and corticosterone at week 7 and week 11 (**C,D**, respectively) in BALB/c mice. Quercetin was administered by a diet containing 1% quercetin (OQ) or intraperitoneal injection (IQ). The control group received the control diet and vehicle only. Values are expressed as mean ± SD. *p < 0.05, **p < 0.01, ***p < 0.001 denote significant differences from the control group; #*p* < 0.05, ##*p* < 0.01, ###*p* < 0.001 denote significant differences from the CDDP group.

To determine the activation of the HPA axis, we first determined the cortisol and corticosterone contents in the plasma. These two glucocorticoids are present in the plasma in rodents with closely correlated dynamics under stress conditions, while corticosterone is considered the main glucocorticoid involved in regulation of chronic stress responses (5, 23). The results showed that compared to the control group, CDDP alone significantly increased the cortisol and corticosterone levels in the plasma. In agreement with the previous study (23), the increases in corticosterone level induced by CDDP were more marked than that of cortisol; it was about 8 times and 5 times higher compared to the control group at week 7 and week 11, respectively (Figure 3). Combined treatment with OQ, IQ, or TAK779 showed a trend of decreasing cortisol and corticosterone levels. However, only IQ significantly attenuated the effects of CDDP in all determiners (*p* < 0.05). We determined the level of cortisol and corticosterone at different time points because the blood sample quantity was not enough for two assays.

**Figure 4:**
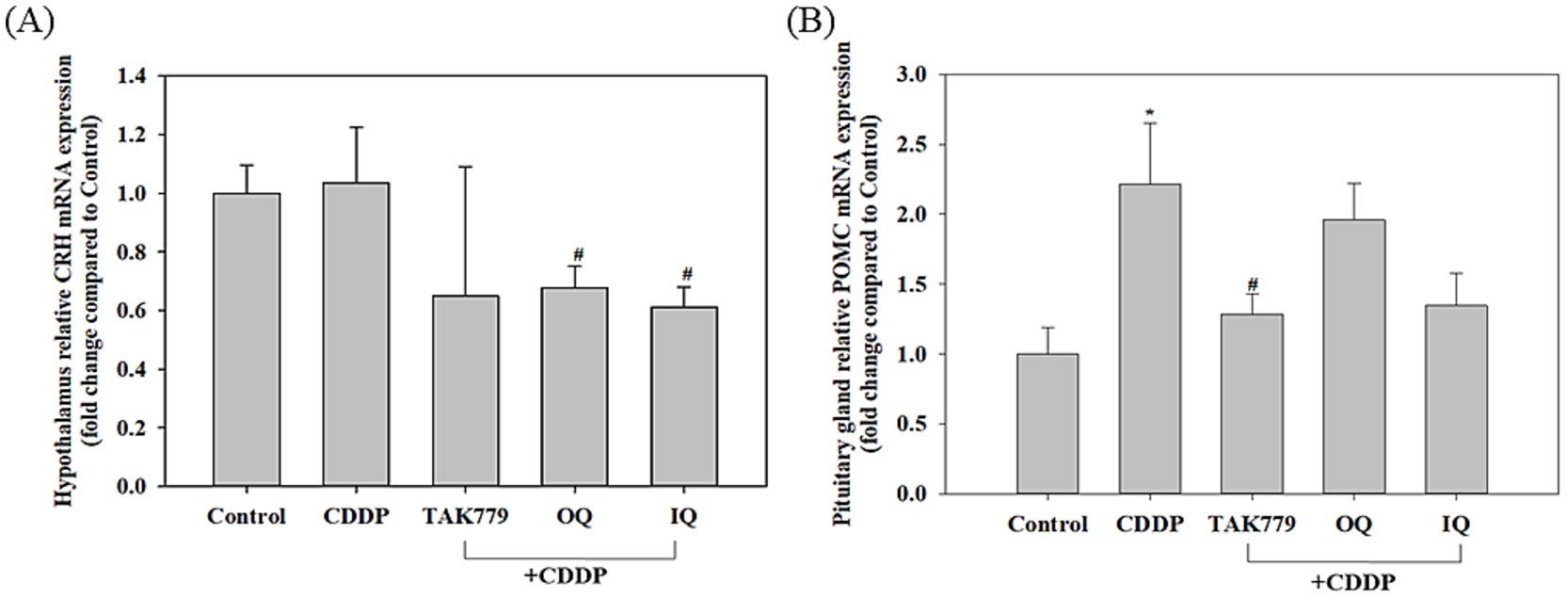
The effect of cisplatin (CDDP) alone or in combination with quercetin or TAK779 on the mRNA expression of CRH **(A)** in the hypothalamus and of POMC **(B)** in the pituitary gland in BALB/c mice. Quercetin was administered by a diet containing 1% quercetin (OQ) or intraperitoneal injection (IQ). The control group received the control diet and vehicle only. Values are expressed as mean ± SD. *p < 0.05, **p < 0.01, ***p < 0.001 denote significant differences from the control group; #*p* < 0.05, ##*p* < 0.01, ###*p* < 0.001 denote significant differences from the CDDP group.

We further examined the mRNA expression of CRH and POMC (the precursor of ACTH). They are two other key hormones related to the activation of the HPA axis and are released by the hypothalamus and pituitary, respectively (6). As shown in Figure 4, compared to the control group, CDDP significantly increased POMC mRNA expression in the pituitary, although only slightly increased CRH mRNA expression in the hypothalamus. In contrast, OQ, IQ, and TAK 799 tended to decrease the mRNA expression of CRH and POMC; however, only OQ and IQ significantly reduced the expression of CRH mRNA (*p* < 0.05) and only TAK779 significantly decreased POMC mRNA expression compared to the CDDP alone group (*p* < 0.05).

### Expression of MCP-1 and CCR2 in the plasma or brainstem tissue

**Figure 5:**
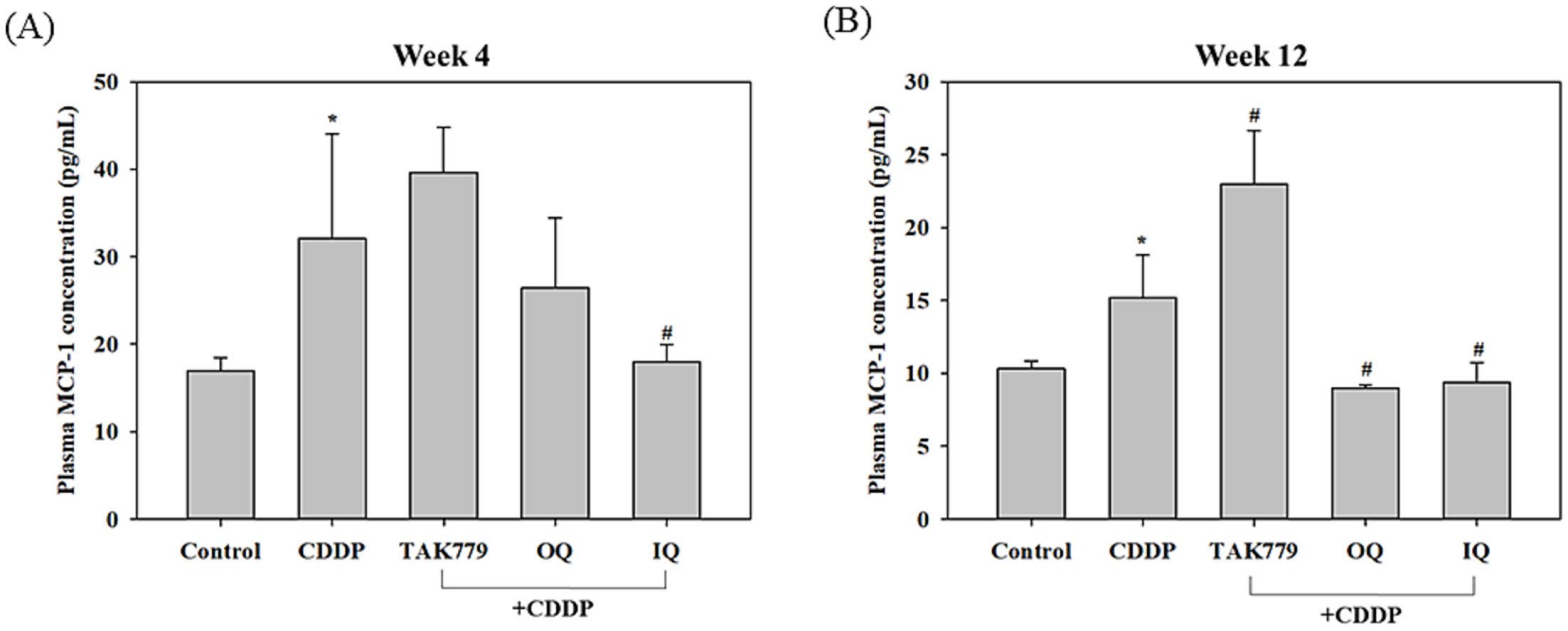
The effect of cisplatin (CDDP) alone or in combination with quercetin or TAK779 on the plasma levels of MCP-1 at week 4 **(A)** and week 12 **(B)** in BALB/c mice. Quercetin was administered by a diet containing 1% quercetin (OQ) or intraperitoneal injection (IQ). The control group received the control diet and vehicle only. Values are expressed as mean ± SD. *p < 0.05, **p < 0.01, ***p < 0.001 denote significant differences from the control group; #*p* < 0.05, ##*p* < 0.01, ###*p* < 0.001 denote significant differences from the CDDP group.

**Figure 6:**
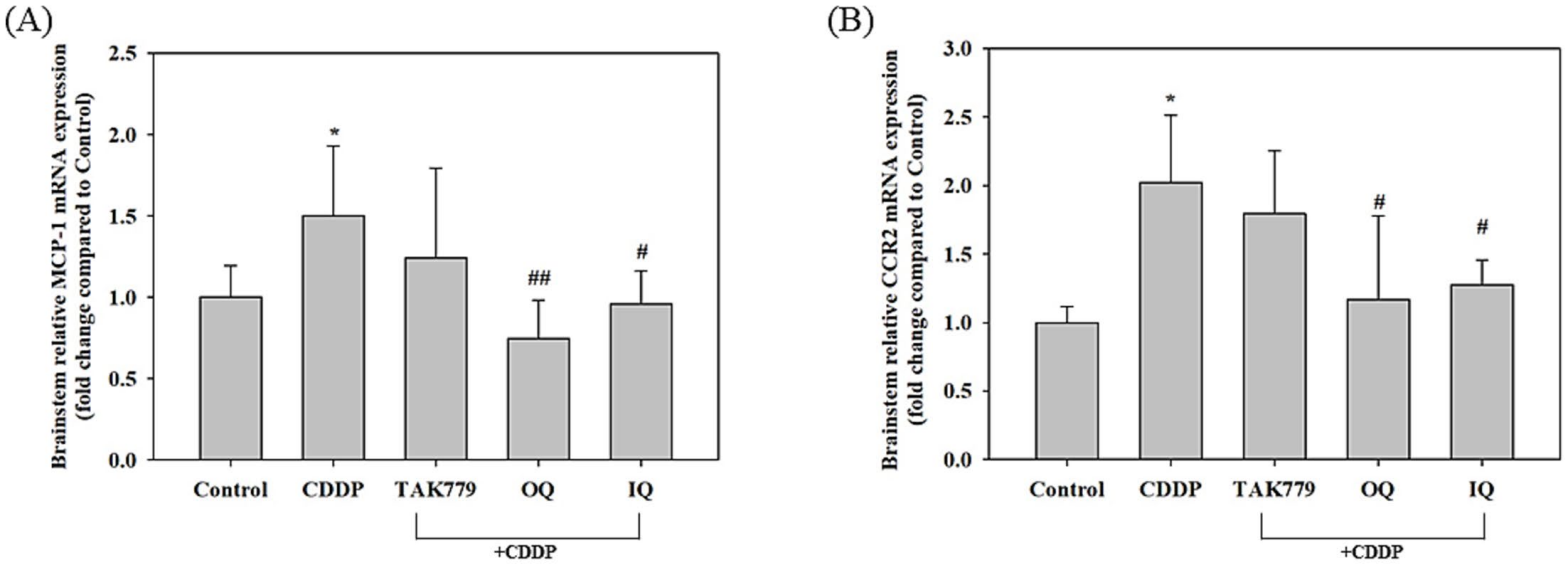
The effect of cisplatin (CDDP) alone or in combination with quercetin or TAK779 on the mRNA expression of MCP-1 **(A)** and CCR2 **(B)** in the brainstem tissues in BALB/c mice. Quercetin was administered by a diet containing 1% quercetin (OQ) or intraperitoneal injection (IQ). The control group received the control diet and vehicle only. Values are expressed as mean ± SD. *p < 0.05, **p < 0.01, ***p < 0.001 denote significant differences from the control group; #*p* < 0.05, ##*p* < 0.01, ###*p* < 0.001 denote significant differences from the CDDP group.

As shown in Figure 5, administration of CDDP alone significantly increased the MCP-1 content in the plasma at week 4 and 12, (*p* < 0.05). OQ and IQ tended to decrease CDDP-induced MCP-1 levels, especially IQ showing a significant decrease at week 4 and 12 (*p* < 0.05). However, combined treatment with TAK779 showed a trend of increasing MCP-1 levels. Furthermore, CDDP significantly increased the expression of MCP-1 and its receptor CCR2 mRNA by approximately 48 and 90%, respectively, in the brainstem tissue of mice (Figure 6). OQ and IQ significantly reduced the induction effect of CDDP on MCP-1 mRNA (*p* = 0.003 and *p* = 0.019, respectively) and CCR2 mRNA (*p* = 0.041 and *p* = 0.023, respectively) whereas TAK779 had no such an effect.

### Proinflammatory cytokines and total quercetin levels in cerebrum tissue

**Figure 7:**
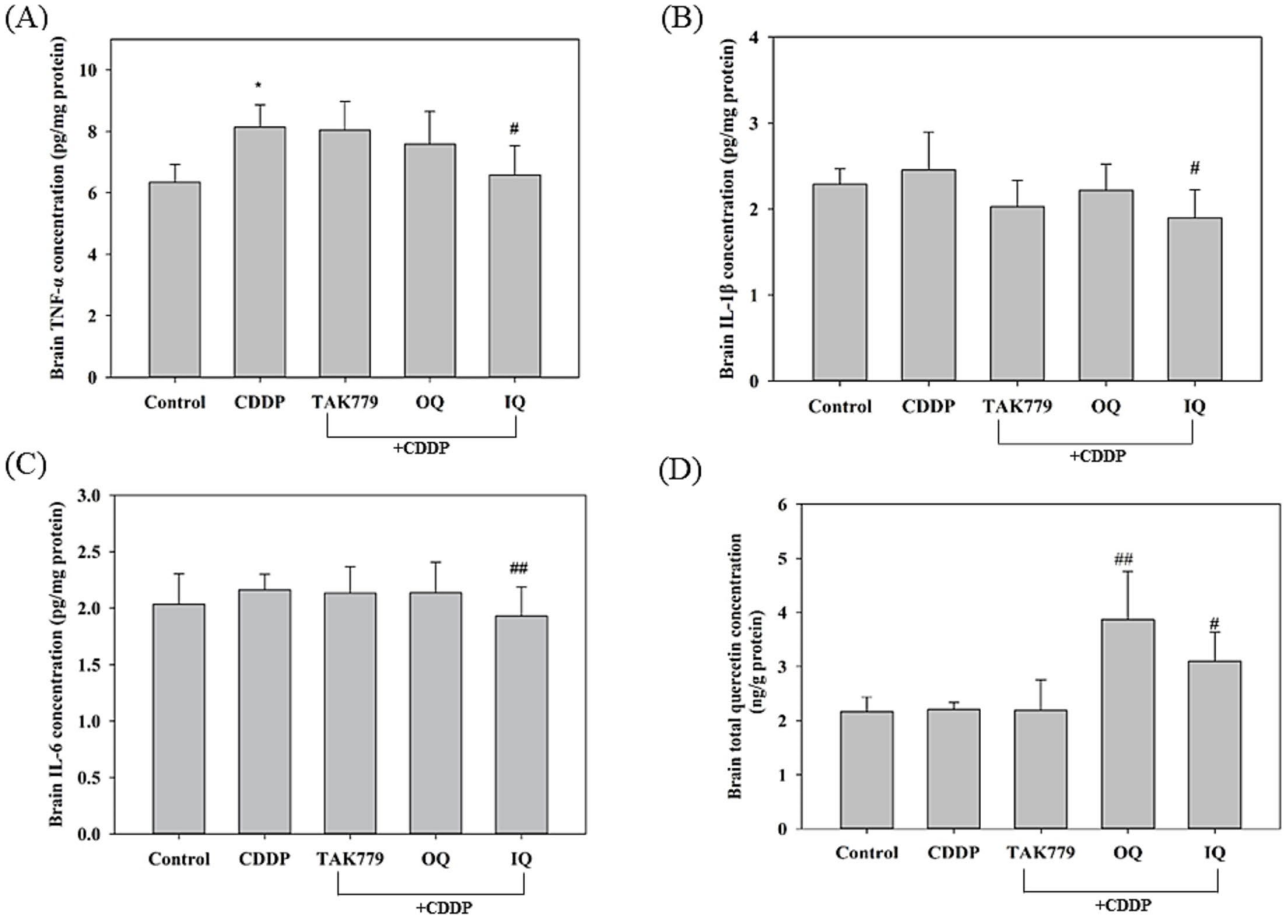
The effect of cisplatin (CDDP) alone or in combination with quercetin or TAK779 on the levels of TNF-*α* **(A)**, IL-1β **(B)**, and IL-6 **(C)** and total quercetin **(D)** in the cerebrum tissues in BALB/c mice. Quercetin was administered by a diet containing 1% quercetin (OQ) or intraperitoneal injection (IQ). The control group received the control diet and vehicle only. Values are expressed as mean ± SD. *p < 0.05, **p < 0.01, ***p < 0.001 denote significant differences from the control group; #*p* < 0.05, ##*p* < 0.01, ###*p* < 0.001 denote significant differences from the CDDP group.

We determined the content of pro-inflammatory cytokines TNF-*α*, IL-1β and IL-6 as well as total quercetin in the cerebrum tissues (Figure 7). Compared to the control group, administration of CDDP significantly increased the TNF-α level (*p* < 0.05). IQ but not OQ or TAK779 significantly decreased the levels of TNF-α, IL-1β, and IL-6 compared with the CDDP group (*p* < 0.05). Using HPLC to analyze the total quercetin (including quercetin and its glucuronide/sulfate conjugated metabolites) content in cerebrum tissue, the results showed that both OQ and IQ significantly and similarly (OQ vs. IQ, *p* = 0.139) increased the total quercetin content in brain tissue (Figure 7D) suggesting that quercetin or its metabolites can indeed cross the blood–brain barrier and enter the brain.

## Discussion

Despite advancements in cancer treatments, chemotherapy is still one approach needed for patients with cancer in many conditions. CDDP is a common chemotherapy used in various cancers. However, its non-specific cytotoxicity results in various side effects such as CRF (24), characterized by persistent fatigue and cannot be alleviated by rest (25). Previous animal studies have shown that CDDP induces fatigue, as evidenced by reducing voluntary wheel running (26), locomotor activity, and grip strength (14). Consistently, the present study also showed that CDDP significantly reduced locomotor activity and grip strength in mice in a time-dependent manner. The supplementation of OQ, IQ, or TAK779 restored the effects of CDDP, although the impact of TAK779 on improving locomotor activity was not significant.

Our previous research indicated the potential of quercetin to alleviate CRF (14) in part through decreased CDDP-induced muscle atrophy. The present study supported the findings. However, our previous study showed that quercetin given by gavage only slightly rather than significantly improved MGS, while OQ and IQ used in the present study significantly improved MGS in mice exposed to CDDP. The different doses and routes (oral vs. intraperitoneal injection) used in the two studies may be the reason for the different results. In the previous study (14), quercetin was administered at a dose of 200 mg/kg body weight daily by oral gavage. In the present study, quercetin was given by a diet containing 1% quercetin in the OQ-supplemented group. That is about 2000 mg/kg (the food intake of the mice was about 5 g/d). The dose was higher than that used in the previous study. Regarding the IQ-supplemented group, quercetin was given by intraperitoneal injection, 10 mg/kg, 3 times per week. Although the dose was lower than the oral doses used, it has been demonstrated that the bioactivity of IQ is better than OQ because of the metabolic conversion of OQ (22). However, more studies are warranted to investigate the effective doses of quercetin given orally. Furthermore, the present study determined the possibility that quercetin and its metabolites also exerted their effects to modulate the disorder of the central nervous system induced by CDDP. As we have mentioned above, several studies show that quercetin could improve hypothalamic–pituitary–adrenal (HPA) axis dysfunction and inflammatory states and exert antidepressant and antianxiety activities (16, 17). Herein, we demonstrated that oral and injected quercetin could attenuate CDDP-induced expression of key factors in the HPA axis and in MCP-1/CCR2 signaling, two important central nervous system factors associated with CRF (4, 11, 27).

It has been shown that a significant and persistent increase in oxidative stress and cortisol concentration, known as stress hormones, are associated with CRF in patients receiving chemotherapy (8). Higher cortisol levels are positively correlated with the severity of physical fatigue experienced in breast cancer patients undergoing chemotherapy or radiotherapy (28). The present study demonstrated that the CRF suppressed effect of quercetin was associated with regulation of the HPA axis because OQ and IQ reduced CDDP-induced plasma cortisol/corticosterone levels, especially IQ. OQ and IQ also tended to reduce the mRNA expression of two other key components of the HPA axis activation, CRH and POMC, in mice exposed to CDDP, although the effects on POMC were insignificant. In agreement with our study, quercetin has been shown to attenuate traumatic brain injury anxiety-like behavior by decreasing the activation of HPA axis, high levels of corticosterone and ACTH (16).

In addition, downregulation of CDDP-induced MCP-1/CCR2 expression might also contribute to the protective effects of OQ/IO on CDDP-induced CRF (11). Our finding is consistent with the study by Nanua et al. (29), which shows that quercetin blocks airway epithelial cell MCP-1 expression. The positive correlation of plasma MCP-1 concentration and CRF have been demonstrated in early breast cancer patients after chemotherapy (30) and mice treated with fluorouracil (5-FU; 31). The animal studies by Weymann et al. (10) showed that combined administration of multiple chemotherapeutic drugs (cyclophosphamide, adriamycin, and 5-FU) increases the expression of MCP-1 and other proteins (TNF-*α*, IL-6, IL-1) in the hypothalamus when the animals exhibited fatigue and drowsiness. Furthermore, a study demonstrated that an antagonist of CCR2 significantly improves CRF and decreases the expression of certain CRF-related central nervous system factors induced by 5-FU (31). Our study also demonstrated that TAK779, an antagonist of CCR2, also partly improved CDDP-induced adverse effects.

It has been shown that quercetin could be distributed to brains in rats and pigs after oral administration (12, 32). Our study also indicated that quercetin or its metabolites can cross the blood–brain barrier and enter the brain in mice because both IQ and OQ increased the accumulation of total quercetin similarly. The dose of OQ used in the study was higher than IQ, however, the efficacy of OQ seemed similar or less than IQ. We speculated that the reasons for such a situation are associated with more conjugated metabolites converting *in vivo* while quercetin is given through oral administration (33). Quercetin and its metabolites possess various biological activities, including antioxidant activity (12, 13). Studies have shown that cisplatin increases oxidative stress (34, 35), contributes to muscle atrophy, and the production of corticosterone and MCP-1 (8, 36, 37), while antioxidants, such as vitamin E, and N-acetyl cysteine, suppress the levels of corticosterone or MCP-1 induced by oxidative stress (36, 37). Therefore, we speculated the mechanisms by which OQ and IQ exerted their effects on regulation of the HPA axis and MCP-1 signaling may be related to its antioxidant activity. However, more studies are needed to investigate this speculation.

An interesting finding in our study is that the antagonist of CCR2, TAK779, also downregulated CDDP-induced POMC (Figure 4) and cortisol/corticosterone at week 4 and week 7, respectively (Figure 3), suggesting the possible impact of MCP-1/CCR2 signaling on the HPA axis. The study by Thompson et al. (38) supports our findings, which shows that animals lacking MCP-1 (MCP-1−/− mice) had significantly lower corticosterone and ACTH expression levels in response to LPS induction compared to normal mice. However, we found that TAK779 did not decrease the plasma level of MCP-1 as well as the mRNA expression of MCP-1 and CCR2. We speculated that the phenomenon is due to TAK-779 inhibiting MCP-1/CCR2 signaling by blocking ligand binding to murine CCR2 and rather than modulating their expression (18). The precise mechanisms by which MCP-1/CCR2 signaling regulated the HPA axis remain unclear. The induction of the proinflammatory cytokines by MCP-1/CCR2 signaling cascade in the brain (39) might play a part of the role because it has been pointed out that the release of proinflammatory cytokines induced by the cancer and/or its treatments into the central nervous system can stimulate the HPA axis (40, 41). However, our study showed that only the suppressed effects of IQ on TNF-*α*, IL-1β, and IL-6 levels in the cerebrum tissue in mice exposed to CDDP were significant, suggesting there may be other mediators involved in the mechanisms. In addition, we only determined the proinflammatory cytokine levels at the end of the study, so whether this affects the results is unclear. More studies are warranted to investigate these issues.

There are some limitations in this study. First, because of a shortage of manpower and insufficient quantity of blood samples, we determined the maximum grip strength and the expression of cortisol/corticosterone at different weeks. Second, we only determined the mRNA expressions of MCP-1 and CCR2 in the plasma or in the brainstem tissue rather than the binding of these two proteins or the downstream factors of MCP-1/CCR2 signaling, the evidence of the activation of this signaling. However, our study still consistently showed a connection between CRF and the expressions of cortisol/corticosterone and MCP-1/CCR2 among groups.

## Conclusion

**Figure 8:**
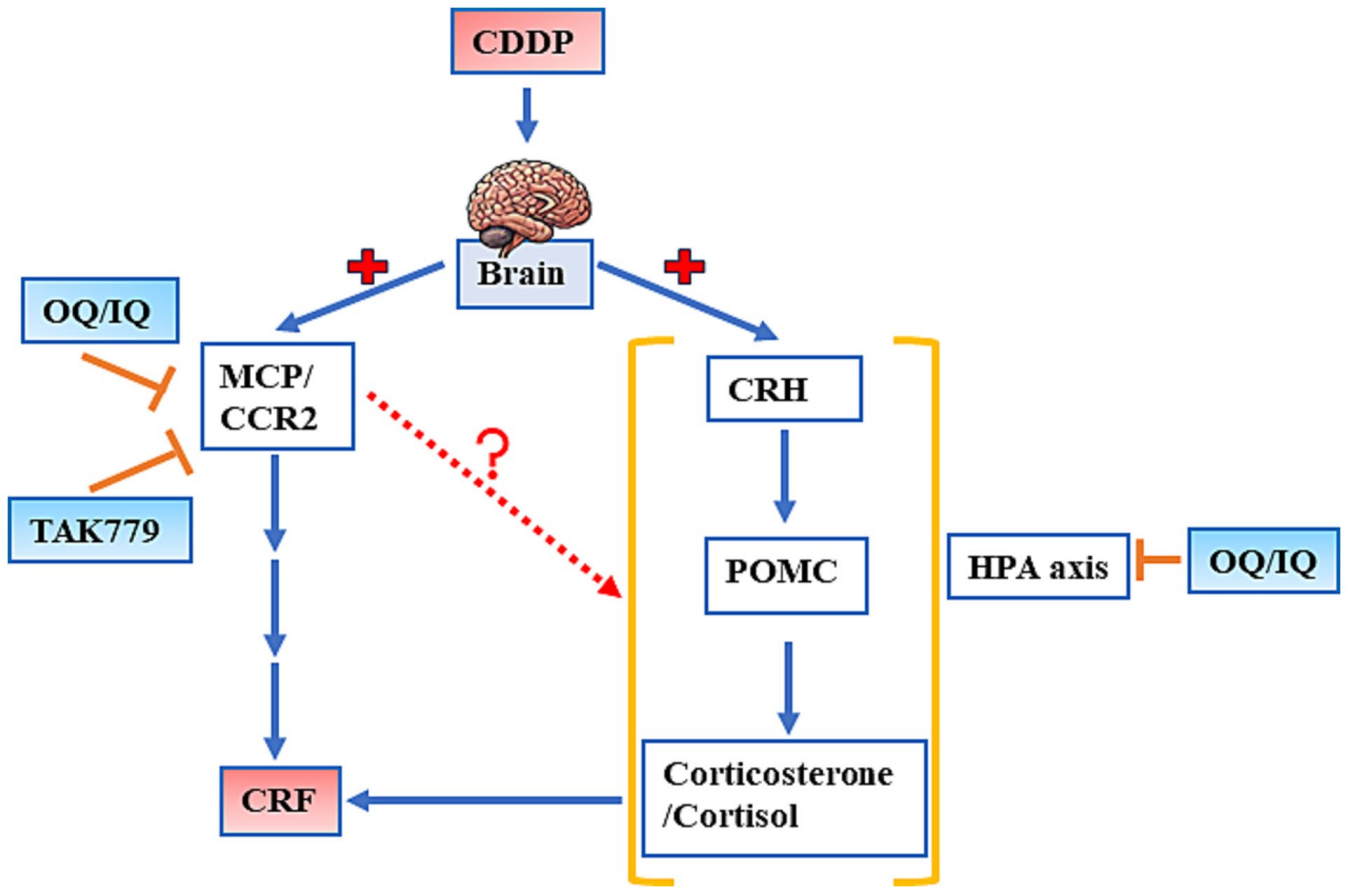
The schematic diagram of the possible mechanisms by which quercetin administered by a diet (OQ) or intraperitoneal injection (IQ) and TAK 779 attenuates cisplatin-induced cancer-related fatigue. CCR2, human CC chemokine receptor R2; CDDP, cisplatin; CRF, cancer-related fatigue; CRH, corticotropin releasing hormone; HPA axis, hypothalamic–pituitary–adrenal axis; MCP-1, monocyte chemoattractant protein-1; POMC, proopiomelanocortin.

Our study suggests that quercetin supplementation mitigates CDDP-induced CRF in mice, as evidenced by improvements in grip strength and locomotor activity, partly by modulating the expression of MCP-1/CCR2 and the key hormones in the HPA axis. In addition, blocking MCP-1/ CCR2 signaling by TAK779 also downregulates the expression of two hormones in HPA axis, POMC and cortisol/corticosterone, suggesting the possible interaction between these two pathways (Figure 8). Further research is needed to elucidate the potential of quercetin as a nutritional supplement for managing CRF in cancer patients undergoing chemotherapy.

## Data Availability

The raw data supporting the conclusions of this article will be made available by the authors, without undue reservation.
